# Magnesium Oxide (MgO) pH-sensitive Sensing Membrane in Electrolyte-Insulator-Semiconductor Structures with CF_4_ Plasma Treatment

**DOI:** 10.1038/s41598-017-07699-3

**Published:** 2017-08-03

**Authors:** Chyuan-Haur Kao, Chia Lung Chang, Wei Ming Su, Yu Tzu Chen, Chien Cheng Lu, Yu Shan Lee, Chen Hao Hong, Chan-Yu Lin, Hsiang Chen

**Affiliations:** 1 Department of Electronic Engineering, Chang Gung University, Taoyuan, 333 Taiwan, ROC; 2 Department of Applied Materials and Optoelectronic Engineering, National Chi Nan University, Puli, 545 Taiwan, ROC; 3 Kidney Research Center, Department of Nephrology, Chang Gung Memorial Hospital, Chang Gung University, College of Medicine, Taoyuan, Taiwan, ROC; 4 Department of Electronic Engineering, Ming Chi University of Technology, New Taipei City, Taiwan, ROC

**Keywords:** Ion transport, Biomedical engineering

## Abstract

Magnesium oxide (MgO) sensing membranes in pH-sensitive electrolyte-insulator-semiconductor structures were fabricated on silicon substrate. To optimize the sensing capability of the membrane, CF_4_ plasma was incorporated to improve the material quality of MgO films. Multiple material analyses including FESEM, XRD, AFM, and SIMS indicate that plasma treatment might enhance the crystallization and increase the grain size. Therefore, the sensing behaviors in terms of sensitivity, linearity, hysteresis effects, and drift rates might be improved. MgO-based EIS membranes with CF_4_ plasma treatment show promise for future industrial biosensing applications.

## Introduction

Intensive studies have been conducted to the development of ion-sensitive field-effect transistors (ISFETs) or electrolyte-insulator-semiconductor (EIS) sensors within the last forty decades. Since the first ISFET was invented by Bergveld in 1970^1^ and the first pH-ISFET was proposed by Caras and Janato^2^, various kinds of ISFET-based biochemical sensors have been evolved. With the advantages of low cost, fast response, and small size, the ISFET-based devices are promising for future clinical biotests or environmental hazard monitoring^3^. However, for the sensing material is one of the key factors in the device sensing applications, there are still some material related problems that may cloud future development of the ISFET-based sensors such as dangling bonds on the sensing membrane and insufficient isolation between the chemical solution/device interface^4^. Since the sensing membrance in the ISFET-based devices plays a major role in the sensing performance, new materials, alternative processes or unconventional treatment have been tried to boost the sensing behaviors. Among novel materials, Nb_2_O_5_^5^, CeO_2_^6^, and Sm_2_O_3_^7^ have been demonstrated as good sensing film materials and the experimental data approve that the ISFET-based sensors incorporating some materials can achieve excellent sensing capability. Futhermore, on the other hand, zooming in the chemical solution/membrance interface can study the subtle membrance material morphologies and characteristics of different materials and gain insight to the interactions between the ions and the surface of various kinds of materials^8^. Not only can the research create high-performance biosensors but help researchers to explore the surface chemical reactions of these novel materials for future applications so as to kill two birds (biosensors creation and material surface characterizations) with one stone^9^. The sensing membrane material is made of metal oxides, which represent an important group of materials exhibiting a wide range of material properties from insulators to semiconductors and conductors^10^. In this research, we select magnesium oxide (MgO), which has been used as ferroelectric materials^11^, building materials^12, 13^, optical materials^14^, and medicine for versatile applications, as the sensing membrane material. MgO with a high bandgap of 7.8 eV and perovskite structures^15^ has also been found in diverse applications including batteries^16^, cosmetics^17^, and catalysts^18^. Until now, MgO-based magnetic field sensors^19^, humidity sensors^20^ and gas sensors^21^ have been proposed and intensively investigated. However, MgO-based chemical liquid solution sensors have not been clearly reported yet.

In this study, we successfully fabricated MgO-based EIS biosensors with a pretty high pH sensitivity of 61 mV/pH. Furthermore, CF_4_ plasmas was used to shape nanostructures^22^ and stabilize the material properties of the MgO membrane. Results indicate that formation of nanostructures can enlarge the contact area and hence optimize the sensing performance. To characterize the influence of CF_4_ plasma on the MgO membrane, f*i*eld-emission scanning electron microscopy (FESEM), X-ray diffraction (XRD), atomic force microscopy (AFM), and secondary ion mass spectroscopy (SIMS) were conducted. Moreover, pH sensitivity, linearity, hysteresis, and drift rates were measured to study the sensing behaviors. These comprehensive studies of the MgO-based EIS biosensors investigate the biochemical sensing properties of MgO-based biosensors and the surface material reaction of the MgO membrane. Furthermore, possible integration of MgO-based or MgO composite material-based devices may be developed, stemming from the solution/MgO interface research.

## Results and Discussion

### Physical Properties

**Figure 1:**
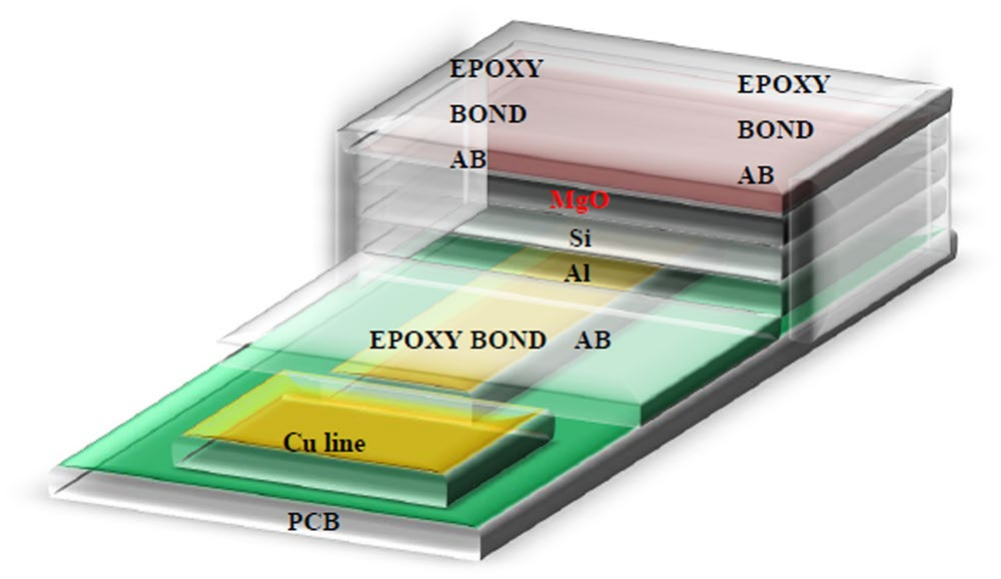
MgO EIS structure.

**Figure 2:**
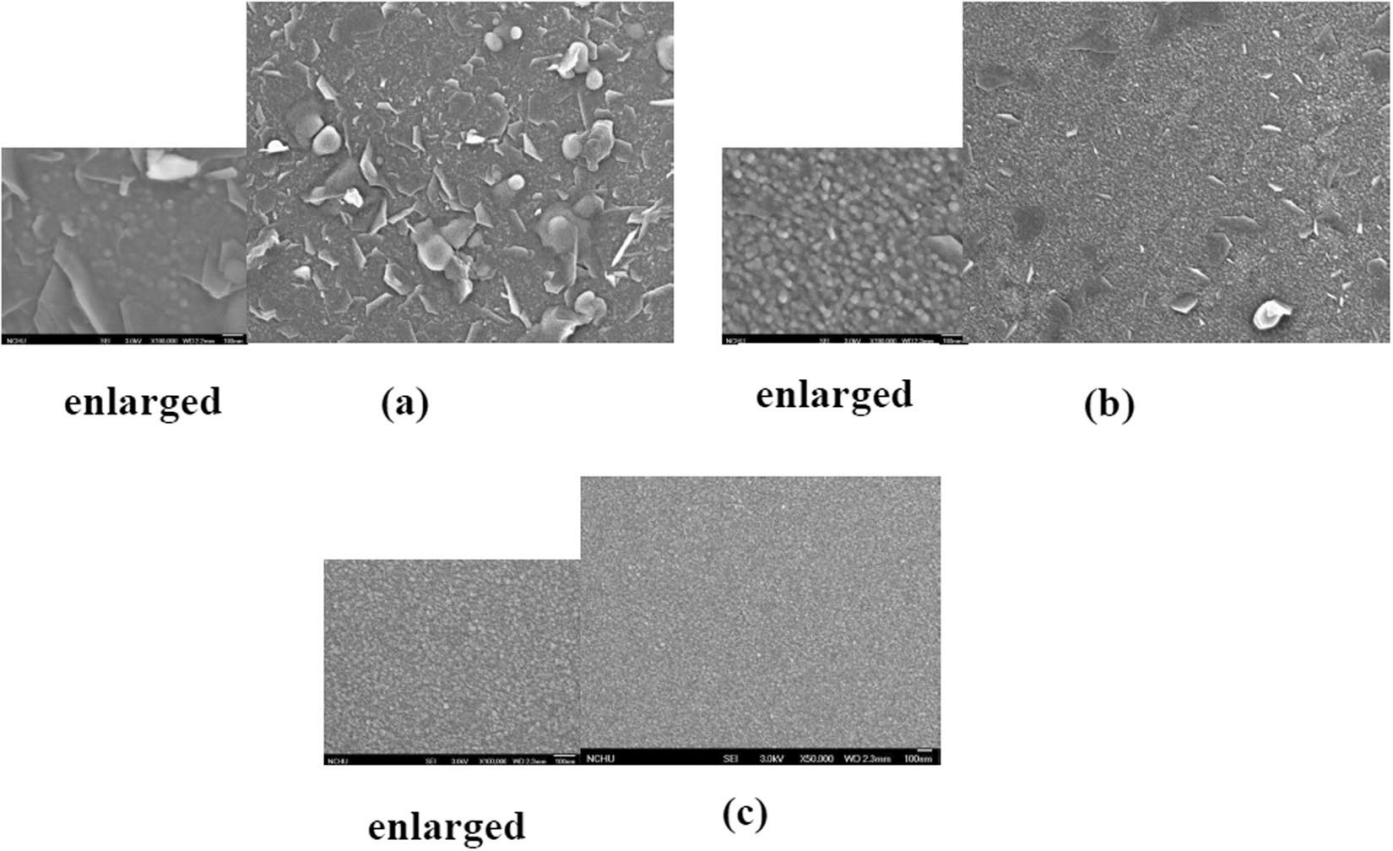
FESEM images of MgO film surfaces for (**a**) the as-deposited sample (**b**) thermal annealed sample (**c**) the sample with CF_4_ plasma treatment. (The scale of the normal FESEM images (**a**) and (**b**) is the same as (**c**)).

The schematic structure of a MgO-based EIS-biosensor is shown in Fig. 1. To characterize the surface of the MgO membrane treated with CF_4_ plasma, multiple material analysis techniques including FESEM, XRD, AFM and SIMS were used. MgO, an alkaline earth oxide, is abundant on earth and incorporating MgO as the membrane may lower the cost of fabrication of biosensors in future industrial applications. A study of surface morphology of the MgO film was realized by FESEM images as shown in Fig. 2(a),(b) and (c). The image of the as-deposited MgO film as shown in Fig. 2(a) presents that many large random flakes with the size around 0.1 µm were distributed on top of the membrane. As the MgO film was treated with annealing, fewer irregular-shaped flakes were distributed on top of the film as shown in Fig. 2(b). Moreover, as the MgO film was treated with CF_4_ plasma, all the flakes were gone. Instead, only nano-sized grains existed on top of the membrane as shown in Fig. 2(c). Compared with annealing, CF_4_ plasma treatment could effectively form nanograins, which might enhance the sensing performance.

**Figure 3:**
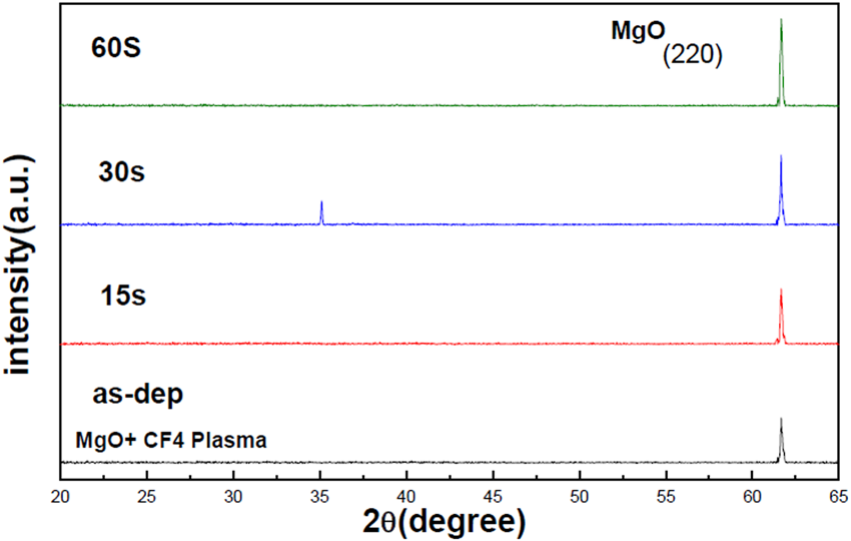
XRD of MgO films with CF_4_ plasma treatment in various conditions.

**Figure 4:**
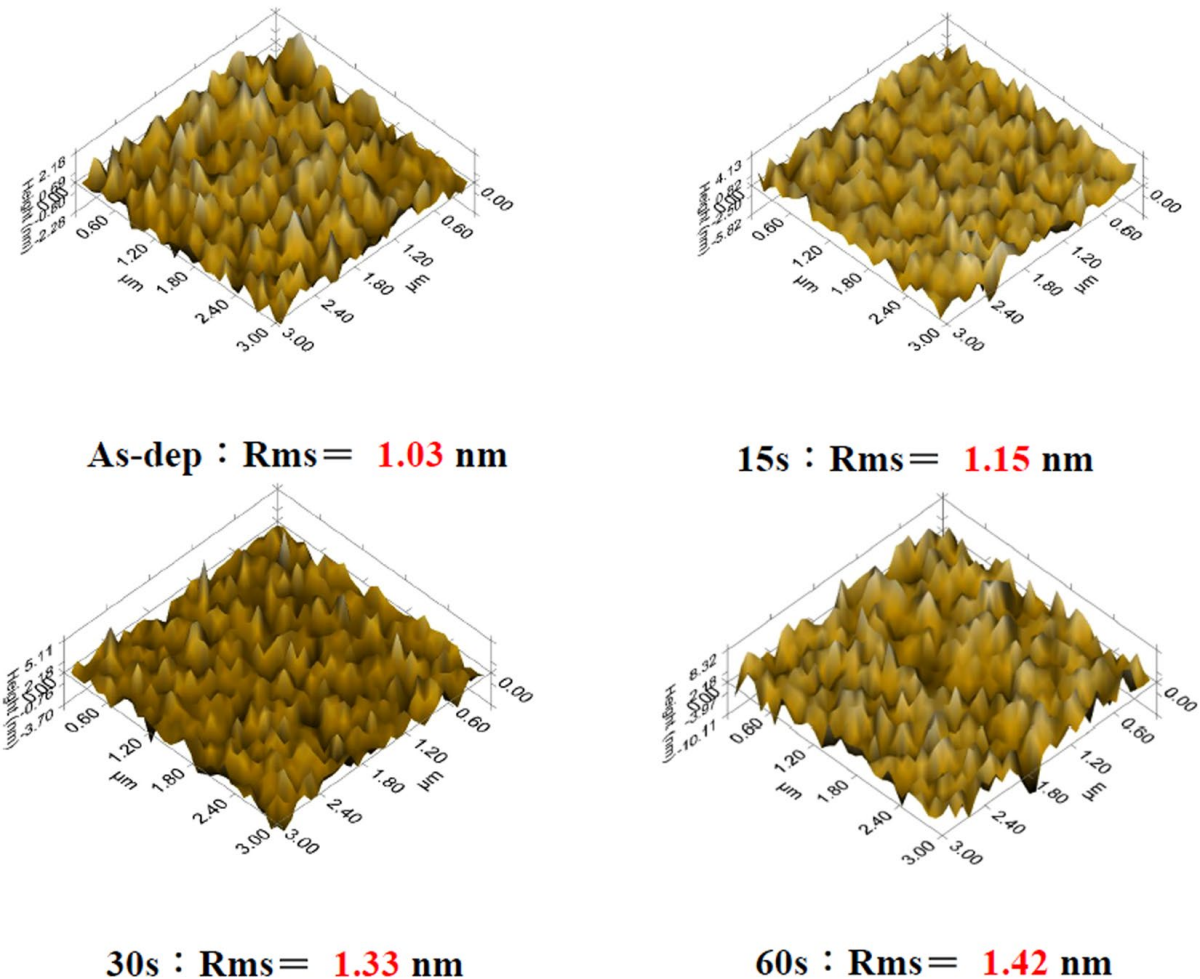
AFM images films with CF_4_ plasma treatment in various conditions.

Since the plasma treatment might be a useful method to boost the sensing properties of the MgO film, XRD, AFM and SIMS were used to evaluate the influence of plasma treatment time on the MgO film. To examine the crystalline structure of the MgO membrane, XRD was used to monitor the MgO crystals with various CF_4_ plasma treatment time. As shown in Fig. 3, as the plasma treatment time increased from 15 sec to 60 sec, the strongest crystallized film occurred at the plasma treatment time of 60 sec. Furthermore, to zoom in the nano-sized grain on top of the surface, AFM analysis was conducted to view the nanograins on top of the membrane as shown in Fig. 4. AFM images reveal the surface scan of the MgO film with no plasma treatment, with plasma treatment time of 15 sec with plasma treatment time of 30 sec, and with plasma treatment time of 60 sec as shown in Fig. 4. AFM analyses indicate the largest size of the MgO nanograins were generated with CF_4_ plasma treatment time of 60 sec. Consistent with the XRD patterns, the largest grains with the strongest crystallization occurred at the plasma treatment time of 60 sec.

**Figure 5:**
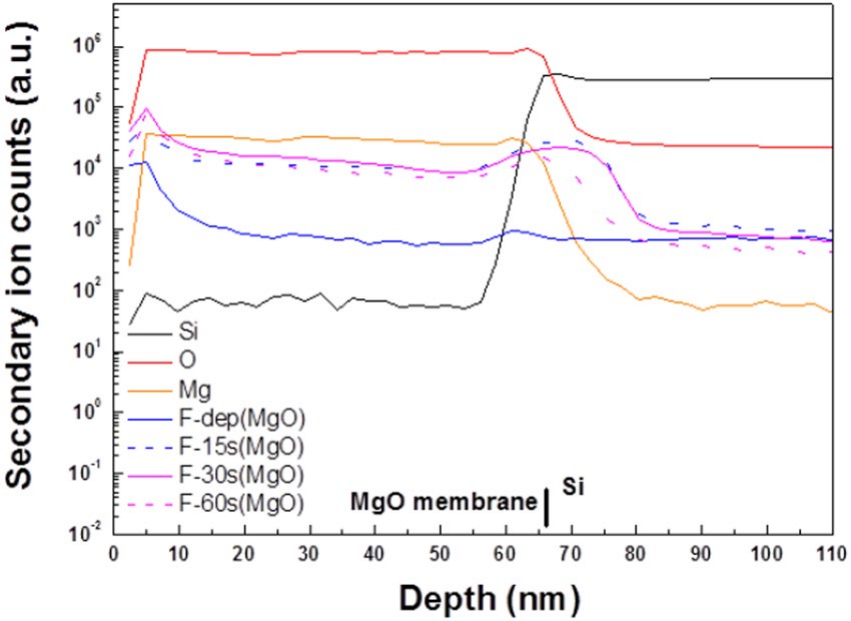
SIMS profiles of MgO sensing membrane/Si with CF_4_ plasma treatment in various conditions.

To gain insight to the influence of fluorine atoms on the MgO film, SIMS was used to detect the fluorine atom distribution inside the MgO/Si film, as shown in Fig. 5. Compared with the as-deposited MgO film, fluorine atom accumulation can be observed near the surface of the MgO film and around the interface of MgO/Si. Since F atoms could bond with dangling bonds and fill the vacancies near the surface and interface, stronger crystallization could be observed for the CF_4_ plasma-treated samples in line with the XRD and AFM analyses. Fixing defects by CF_4_ plasma might enhance the material quality and sensing capability.

### Sensing characterization

After material analyses, the pH sensitive performance of MgO sensing membrane was examined. Sensing performance including pH sensitivity, linearity, hysteresis effects, and drifting voltages before and after plasma treatments could be observed. The sensing parameters were taken by using HP4284A precision LCR meter. To make the surface reaction stable, all the samples were soaked in the RO water for 12 hours. The corresponding voltages in the C-V curves for all the samples were calculated with 0.4 C_max_ as the reference. Then, the threshold voltage changes versus the pH values could be extracted.

Moreover, equation (1)^23^ portrays the flat band voltage of the EIS structure as follows:

1 $${V}_{FB}={E}_{{Re}f}-{{\Psi }}_{0}+{\chi }^{sol}-\frac{{{\Phi }}_{Si}}{q}-\frac{{Q}_{ox}-{Q}_{ss}}{{C}_{ox}},$$

where *E*_*Ref*_ is the reference electrode potential, *χ*^*sol*^ is the surface dipole potential of the solution, *Φ*_*si*_ is the work function of silicon, and *ψ*_0_ is the liquid junction potential difference. All these terms are fixed values except for *ψ*_0_, and it is this term which causes the EIS structure responsive to the tested solution owing to polarization and the formation of the potential barrier, which connects to the quantity or concentration of *H*^+^ ions.

In addition, the site-binding model describes the ionic consumption procedures between the solution/membrane interface^24–26^. The measured voltage on the surface potential (ψ) can be expressed as a function of the membrane and the pH concentration of the electrolyte. The value of (ψ) can be figured out using Eq. (2).

2 $$\psi =2.303\frac{kT}{q}\frac{\beta }{\beta +1}(p{H}_{pzc}-pH)$$

where k is Boltzmann’s constant, T is the temperature, q is the elementary charge, pH_pzc_ is the pH value with no charge, and β is a factor that indicates the sensitivity of the gate membrane. Furthermore, the β is closely connected to the density of surface hydroxyl groups, as described in Eq. (3).

3 $$\beta =\frac{2{q}^{2}Ns\sqrt{{K}_{a}{K}_{b}}}{KT{C}_{DL}}$$

where N_s_ is the number of surface site per unit area and C_DL_ is the double layer capacitance based on the Gouy–Chapman–Stern model^27^.

Apparently, from Equation (3), it is clear that the higher the surface site density N_s_ is, the higher the pH sensitivity and linearity the membrane will have. The surface site density is closely related to the surface roughness and the material quality of the membrane. Varying the pH value of the electrolyte could result in the flat band voltage shift of the C-V curves. Based on the material analyses and the site binding model, CF_4_ plasma treatment could increase the roughness and enhance crystallization. Therefore, the surface site density for the sample with plasma treatment could be larger than the as-deposited sample and hence have higher sensing performance.

**Figure 6:**
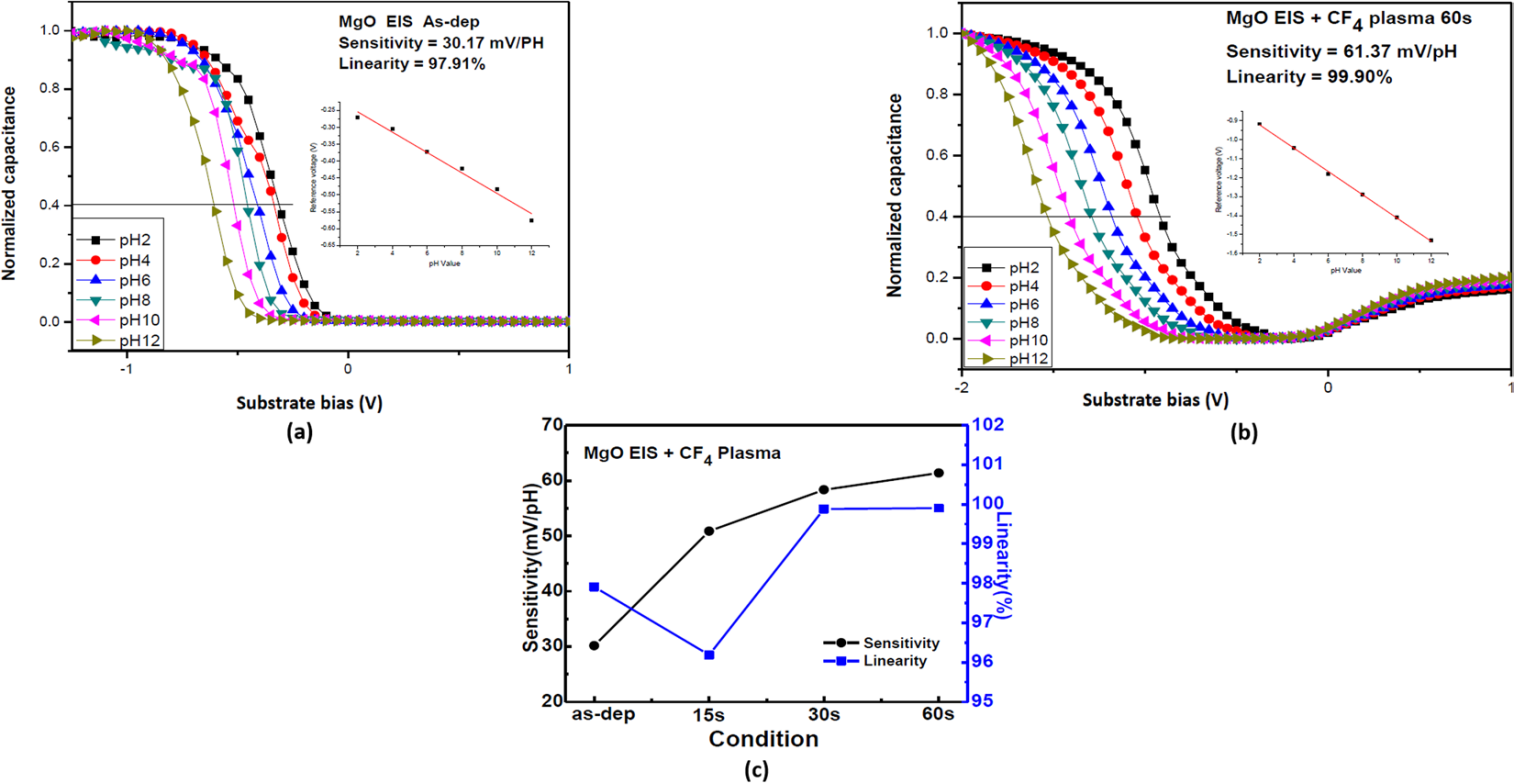
The normalized C-V curve of the sample (**a**) without and (**b**) with CF_4_ plasma treatment for 60 sec. The inset figure represents the sensitivity and linearity. (**c**) The sensitivity and linearity of the samples with CF_4_ plasma treatment in various conditions.

To evaluate the pH sensitivity of the MgO-based EIS device, a set of C-V curves at pH values of 2, 4, 6, 8, 10, and 12 were measured. In addition, the sensitivity and linearity could be calculated from these C-V curves. (We set 0.4 C_max_ as the reference and extract the voltage variation versus the change of the pH value). The normalized C-V curves without CF_4_ plasma treatment is shown in Fig. 6(a) and the normalized C-V curves with plasma treatment for 60 sec are shown in Fig. 6(b). Furthermore, the sensitivity and linearity without plasma treatment and with plasma treatment for 15 sec, 30 sec, and 60 sec calculated from the C-V curves are plotted in Fig. 6(c). Consistent with the material analyses, the MgO membrane with plasma treatment for 60 sec exhibited lager sensitivity of 61.37 mV/pH and linearity of 99.9% than all the other samples, indicating that the membrane with stronger crystallization and larger roughness enhance the pH sensitivity and linearity of the membrane. Moreover, incorporation of fluorine atoms might improve the surface and interfacial material quality and increase the surface site density. Based on recent studies^28, 29^, CF_4_ plasma treatment could generate surface sites. In addition, the increase of the surface sites could transform the surface morphologies as revealed in AFM images as shown in Fig. 4^30^. CF_4_ plasma could cause the membrane fluorinated and hence induce the formation of the fluorine atom related surface sites, which could enhance detection of various ions on the surface^31, 32^. Therefore, the number of the surface sites could be increased and the sensitivity of the membrane with CF_4_ plasma treatment could be enhanced. Therefore, CF_4_ plasma treatment might increase the density of binding sites and enlarge the value of *β*.

**Figure 7:**
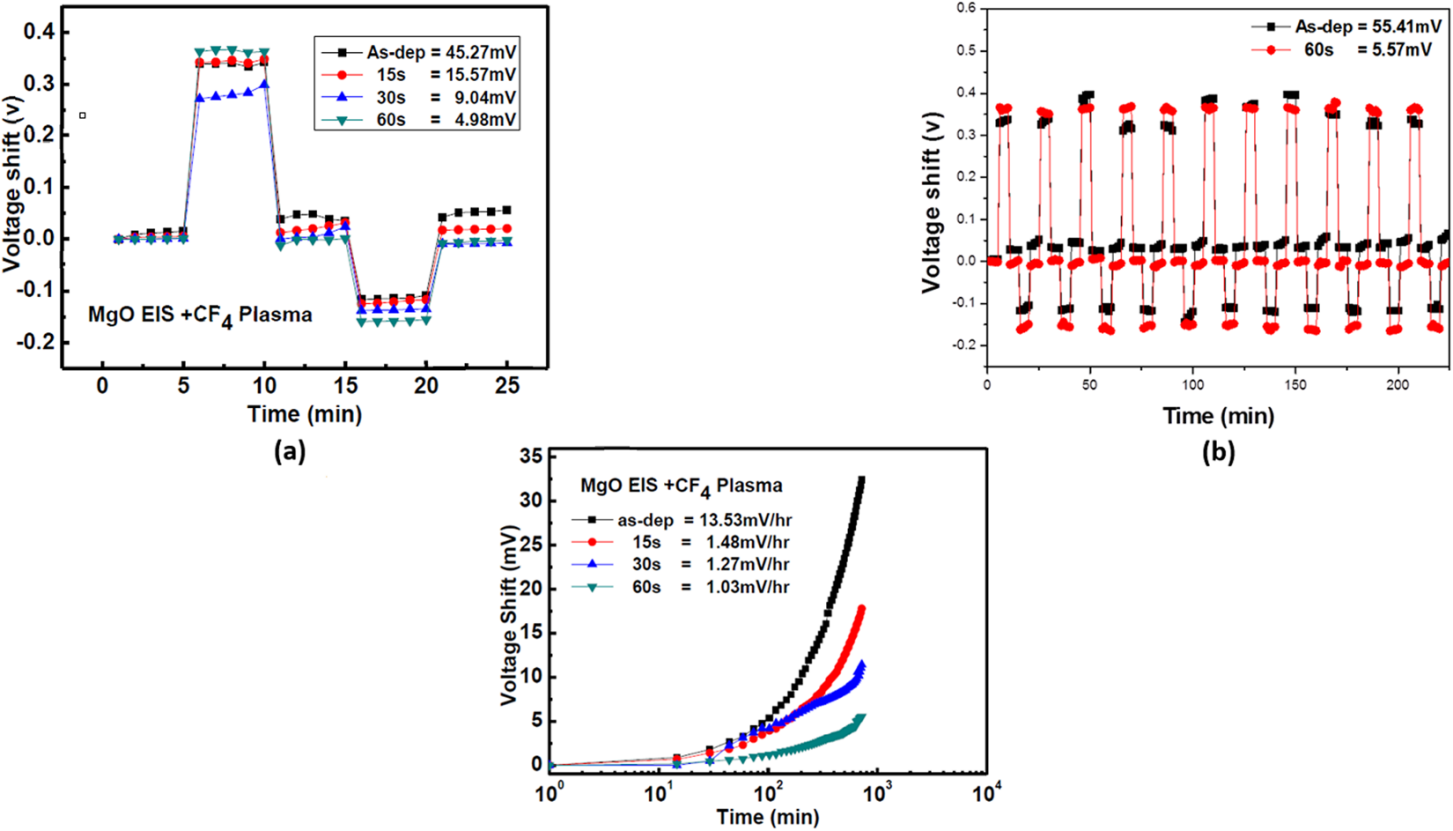
(**a**) The hysteresis of MgO sensing membranes with CF_4_ plasma treatment in various conditions during the pH loop of 7 → 4 → 7 → 10 → 7 over a period of 25 minutes. (**b**) The hysteresis of MgO sensing membranes without CF_4_ plasma treatment and with 60 sec CF_4_ plasma treatment after the pH loop of 11 cycles. (**c**) The drift voltage of MgO sensing membrane with CF_4_ plasma treatment in various conditions, then dipped in pH 7 buffer solution for 12 hours.

Furthermore, the hysteresis effects of MgO sensing membranes prepared under various plasma treatment conditions is shown in Fig. 7(a). The EIS biosensors were submerged in the solutions of the pH loop of 7 → 4 → 7 → 10 → 7. After 11 cycles (7 → 4 → 7 → 10 → 7) of operations as shown in Fig. 7(b), the hysteresis voltage of the sample without treatment (as-deposited) and with CF_4_ plasma for 60 sec increased to 55.51 mV and 5.57 mV, respectively. The plasma treatment could effectively remove the dangling bonds and fix the defects to sustain excellent performance and stability. Since hysteresis effects might be attributed to the dangling bonds of the defect structure of the membrane, these sites could interact with the hydroxyl groups and cause the hysteresis effects. Results indicate that the sample with plasma treatment for 60 sec had the least hysteresis voltage of 4.98 mV, indicating that plasma treatment could effectively remove the dangling bonds and fix the defects. Moreover, the drift voltage of the membrane could be detected by putting the biosensor into the solution of the pH value of 7 for a period of time and measuring the shift of the gate voltage. The drift voltages of the pH-sensitive sensor were measured as shown in Fig. 7(c). Similarly, the sample with CF_4_ plasma treatment for 60 sec had the least drift voltage of 1.03 mV/hr. Since the voltage drift effect could be explained by the hopping and/or trap-limited transport of water-related species, localized defects or dangling bonds might react with the solution of pH value of 7 and cause the drift voltage^7^. Plasma treatment could effectively mitigate the defects and passivate the dangling bonds. Therefore the voltage drift effect could be minimized.

**Figure 8:**
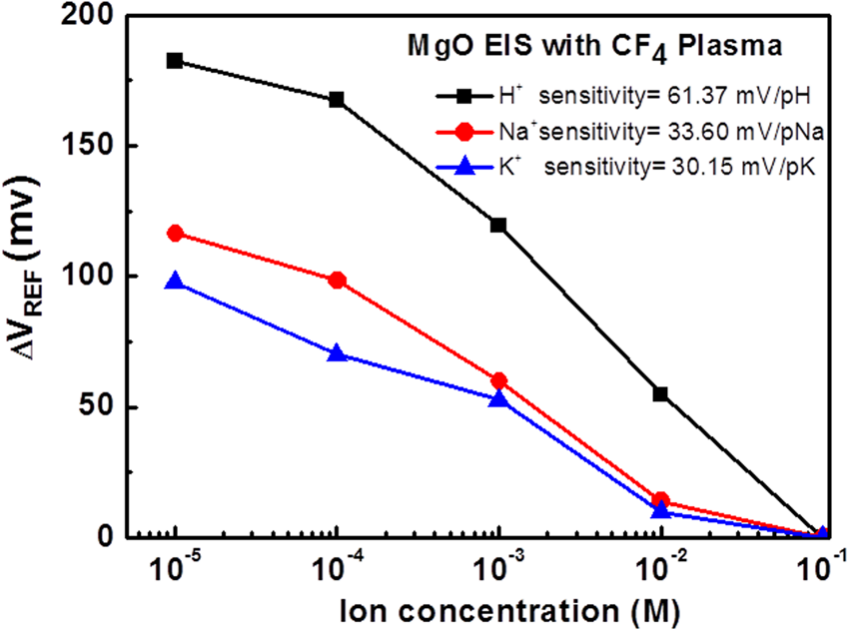
H^+^, Na^+^, and K^+^ ion sensitivities of the membrane with 60 sec CF_4_ plasma treatment.

Finally, to reveal the selectivity of the EIS biosensor, the sensitivity of potassium ions and sodium ions were included in the revised manuscript so that the selectivity among H^+^, Na^+^, and K^+^ could be presented for the EIS biosensor with MgO membrane. At first, we prepared the 5 mM Tris/HCl buffer solution and the pH value of the solution was kept at 8.7. After that, we used the micropipette to control the concentrations of sodium and potassium ions in the range between 10^−5^ to 10^−1^ M with injection of 1 M NaCl/Tris-HCl and 1 M KCl/Tris-HCl into buffer electrolyte, respectively. Then, we picked the as-deposited sample and the sample treated with 60 sec CF_4_ plasma for comparison. The pNa and pK sensitivity of the as-deposited sample are 10.12 mV/pNa and 8.36 mV/pK, respectively. Moreover, the pNa and pK sensitivity of the sample with 60 sec CF_4_ plasma were 33.60 mV/pNa and 30.15 mV/pK, respectively. Furthermore, the sensitivities for H^+^, Na^+^, and K^+^ of the MgO sensing membrane treated with 60 sec CF_4_ plasma was graphed and compared as shown in Fig. 8. It can be seen that the EIS structure with MgO sensing membrane was more responsive to H^+^ ion (61.37 mV/pH) compared with Na^+^ (33.60 mV/pNa) and K^+^ (30.15 mV/pK).

## Conclusions

MgO-based EIS structures grown on Si substrate by RF sputtering were fabricated. Material analyses indicate that incorporating CF_4_ plasma treatment for 60 sec on the MgO membrane could enhance crystallization and increase the size of the nanograin. Furthermore, incorporation of F atoms could fix the defects and improve the material quality of the MgO membrane. Therefore, the pH sensing performance of the MgO membrane could be effectively optimized. Results show that MgO membrane with plasma treatment for 60 sec exhibited larger sensitivity of 61.37 mV/pH and linearity of 99.9%. Moreover, the EIS structure with MgO sensing membrane was more responsive to H^+^ ion compared with Na^+^ and K^+^ ions. MgO-based EIS membranes with CF_4_ plasma treatment show promise for future industrial biosensing applications.

## Methods

The electrolyte-insulator-semiconductor (EIS) structures incorporating MgO sensing membranes were manufactured 4 inch n-type(100) silicon wafers with a resistivity of 5–10 Ω-cm. The wafers were cleaned with a normal RCA process and utilized HF-dip (HF:H_2_O = 1:100) to take away the native oxide before the deposition of MgO films. After the treatment, a 60 nm MgO film was deposited on the Si substrate by radio frequency (RF) sputtered from a magnesium target in diluted O_2_ surroundings (Ar/O_2_ = 25sccm/10sccm), The RF power and the ambient pressure were 150 Watt and 20 mTorr, respectively. After deposition, some wafers were subjected to a post-CF_4_ plasma treatment in a plasma-enhanced chemical vapor deposition (PECVD) system with an RF power of 30 W and a processing pressure of 300 mTorr for 15 sec, 30 sec and 60 sec, respectively. The instrumental type of PECVD was SAMCO PD-220N for the deposition of silicon-based thin films and plasma treatment. Then, the wafers were consequently processed with rapid thermal annealing (RTA) using a standard thermal annealing system under ambient N_2_ condition for 30 sec at 600 °C temperatures. Afterwards, Al film with 300nm-thick was deposited on the back-side contact of the Si chip. The sensing film size was defined through photolithographic processing under a photosensitive epoxy (SU8–2005, Micro-Chem). EIS structures were then attached on the copper lines of a printed circuit board (PCB) by using a silver gel to structure conductive lines. Epoxy package was used to separate the EIS structure and the copper line. The detailed EIS structure was illustrated in Fig. 1.
