# Author Correction: Magnesium Oxide (MgO) pH-sensitive Sensing Membrane in Electrolyte-Insulator-Semiconductor Structures with CF_4_ Plasma Treatment

**DOI:** 10.1038/s41598-020-63702-4

**Published:** 2020-04-17

**Authors:** Chyuan-Haur Kao, Chia Lung Chang, Wei Ming Su, Yu Tzu Chen, Chien Cheng Lu, Yu Shan Lee, Chen Hao Hong, Chan-Yu Lin, Hsiang Chen

**Affiliations:** 1 Department of Electronic Engineering, Chang Gung University, Taoyuan, 333 Taiwan, ROC; 2 Department of Applied Materials and Optoelectronic Engineering, National Chi Nan University, Puli, 545 Taiwan, ROC; 3 Kidney Research Center, Department of Nephrology, Chang Gung Memorial Hospital, Chang Gung University, College of Medicine, Taoyuan, Taiwan, ROC; 4 Department of Electronic Engineering, Ming Chi University of Technology, New Taipei City, Taiwan, ROC

Correction to: *Scientific Reports* 10.1038/s41598-017-07699-3, published online 03 August 2017

In the original version of this Article, Chan-Yu Lin was incorrectly affiliated with ‘Department of Electronic Engineering, Ming Chi University of Technology, New Taipei City, Taiwan, ROC’. The correct affiliation is listed below.

Kidney Research Center, Department of Nephrology, Chang Gung Memorial Hospital, Chang Gung University, College of Medicine, Taoyuan, Taiwan, ROC

This error has now been corrected in the PDF and HTML versions of the Article.

